# Reck-Notch1 Signaling Mediates miR-221/222 Regulation of Lung Cancer Stem Cells in NSCLC

**DOI:** 10.3389/fcell.2021.663279

**Published:** 2021-04-20

**Authors:** Yuefan Guo, Guangxue Wang, Zhongrui Wang, Xin Ding, Lu Qian, Ya Li, Zhen Ren, Pengfei Liu, Wenjing Ma, Danni Li, Yuan Li, Qian Zhao, Jinhui Lü, Qinchuan Li, Qinhong Wang, Zuoren Yu

**Affiliations:** ^1^Research Center for Translational Medicine, Shanghai East Hospital, Tongji University School of Medicine, Shanghai, China; ^2^Jinzhou Medical University, School of Basic Medical, Jinzhou, China; ^3^Dalian Medical University, School of Basic Medical, Dalian, China

**Keywords:** non-small cell lung cancer, cancer stem cell, miR-221/222, Reck, Notch1 signaling

## Abstract

Cancer stem cells (CSCs) contribute to the cancer initiation, metastasis and drug resistance in non-small cell lung cancer (NSCLC). Herein, we identified a miR-221/222 cluster as a novel regulator of CSCs in NSCLC. Targeted overexpression or knockdown of miR-221/222 in NSCLC cells revealed the essential roles of miR-221/222 in regulation of lung cancer cell proliferation, mammosphere formation, subpopulation of CD133^+^ CSCs and the expression of stemness genes including OCT4, NANOG and h-TERT. The *in vivo* animal study showed that overexpression of miR-221/222 significantly enhanced the capacity of lung cancer cells to develop tumor and grow faster, indicating the importance of miR-221/222 in tumorigenesis and tumor growth. Mechanistically, Reck was found to be a key direct target gene of miR-221/222 in NSCLC. Overexpression of miR-221/222 significantly suppressed Reck expression, activated Notch1 signaling and increased the level of NICD. As an activated form of Notch1, NICD leads to enhanced stemness in NSCLC cells. In addition, knockdown of Reck by siRNA not only mimicked miR-221/222 effects, but also demonstrated involvement of Reck in the miR-221/222-induced activation of Notch1 signaling, verifying the essential roles of the miR-221/222-Reck-Notch1 axis in regulating stemness of NSCLC cells. These findings uncover a novel mechanism by which lung CSCs are significantly manipulated by miR-221/222, and provide a potential therapeutic target for the treatment of NSCLC.

## Introduction

Lung cancer is the leading cause of cancer-related deaths worldwide (Chen et al., 2016; Bray et al., 2018). It is classified into two categories including small cell lung cancer (SCLC) and non-small cell lung cancer (NSCLC). The majority of lung cancer cases (∼85%) belong to NSCLC all over the world (Wood et al., 2015; Jiang et al., 2018). Although traditional chemotherapy and molecular targeted therapeutic drugs have been widely applied to NSCLC patients after primary treatment with surgical resection, the 5-year survival rate is still very low mostly due to tumor recurrence and drug resistance (Jiang et al., 2018). It is the small subpopulation of stem-like cells within tumors that are believed to be responsible for the tumor recurrence and drug resistance (Yu et al., 2012). They are called cancer stem cells (CSCs) or tumor initiating cells (TICs), and characterized by self-renewal, differentiation and strong ability of tumor-regeneration.

Since first evidence for CSCs was published in 1997 in leukemia (Bonnet and Dick, 1997), CSC isolation and identification in solid cancers including breast, lung and prostate cancer have been frequently reported. In lung cancer, CSCs are considered as the main reason causing tumor initiation, dormancy, recurrence, metastasis and resistance to therapy (Pardal et al., 2003; Eramo et al., 2010). As such, determination of the molecular mechanism underlying the behavior of lung CSCs and the development of target molecules against lung CSCs could potentially bring great benefits to the patients with lung cancer (MacDonagh et al., 2016). Up to date, several stem cell markers including CD133, ALDH1 and CXCR4 have been successfully applied to isolate lung CSCs from tumor tissues (Wang et al., 2013; Roudi et al., 2015; Eramo et al., 2016; Aghajani et al., 2019). However, the understanding of lung CSCs remains largely unclear.

Non-coding RNAs (ncRNAs), including long non-coding RNAs (lncRNAs) and microRNAs (miRNAs), have been well demonstrated to be crucial for gene expression regulation, as well as epigenetic regulation (Cech and Steitz, 2014; Adams et al., 2017). MiRNAs are a class of single-stranded small RNAs with 18–24 nucleotides in length, usually regulating gene expression at the post-transcriptional level through binding to the 3’UTR of target mRNAs (Krek et al., 2005; Rupaimoole and Slack, 2017). During the development and progression of cancer, miRNAs play important roles in regulating cancer cell stemness, tumor regeneration, cancer cell metastasis and chemo-resistance (Shimono et al., 2009; Bu et al., 2016; Khan et al., 2019). In NSCLC, aberrant expression of miRNAs including miR-582-3p, miR-1246, and miR-1290 have been reported to regulate CSCs and promote tumor progression (Fang et al., 2015; Zhang et al., 2016; Shukla et al., 2018).

In the current study, we performed a miRNA screening analysis on the CD133^+^ CSC subpopulation isolated from both A549 and H1299 NSCLC cell lines, and identified a subset of miRNAs with differential expression in CSCs including upregulation of miR-221/222. Enforced overexpression of miR-221/222 in NSCLC cells enhanced cell proliferation and CSC properties *in vitro*. Xenografting of A549 cells stably expressing miR-221/222 into immune-deficient nude mice developed much bigger tumors than control cells, indicating that miR-221/222 plays an essential role in the process of tumorigenesis and tumor growth. Gene reversion-inducing cysteine-rich protein with kazal motifs (Reck) was identified as a direct target gene of miR-221/222 in NSCLC, which involves in the activation of downstream Notch1 signaling and subsequent self-renewal of lung CSCs. Our findings for the first time demonstrated that miR-221/222-Reck-Notch signaling acts as a critical regulatory mechanism underlying the stemness of CSCs in NSCLC.

## Materials and Methods

### Clinical Tumor Samples

Human NSCLC tumor samples were collected from Tongji University Shanghai East Hospital. All the procedures were approved by the Institutional Review Board (IRB) of Tongji University Shanghai East Hospital. All patients were provided with a written informed consent form.

### Animals

Six-week-old immunodeficient male nude mice were purchased from the Silaike Animal Company (Shanghai, China) for *in vivo* assays. 5 × 10^5^ A549 cells with or without stable expression of miR-221/222 were transplanted into immune-deficient nude mice by subcutaneous injection to establish the NSCLC animal model. All animal studies were performed following the protocols approved by the Institutional Animal Care and Use Committee of Tongji University School of Medicine.

### Cells

Human lung cancer cell lines A549, NCI-H1299 and human bronchial non-tumorigenic epithelial cell line BEAS-2B were originally purchased from ATCC, maintained in our lab, and cultured in Dulbecco’s Modified Eagle’s Medium (DMEM) with 100 mg/L penicillin and streptomycin and 10% fetal bovine serum (FBS). All of these cells were cultured at 37°C in a humidified environment with 5% CO2.

### Vectors, Oligos, and Transfection

miR-221/222-overexpression vector for animal study is a lentivirus-based product from Novobiosci Company (Shanghai, China). Tet-inducible PB-TRE3G vector was used to prepare the tet-on 3G miR-221/222 system by inserting primary miR-221/222 sequence (chrX.c45747283-45746050). 4 μg of PB-TRE3G-miR-221/222 and 3 μg PB-Tet 3G were co-transfected into A549 cells using lipofectamine 2000 (Invitrogen, United States) cultured in the tet-free medium. In 24 h, doxycycline (Boyotime, Shanghai, China) was added into the medium (Dox+, 3 μg/ml). Another dish in parallel without treatment with doxycycline (Dox-) was used for control. Cells were collected for RNA and protein isolation in 48 h after doxycycline treatment. miRNA mimics, anti-miRNA inhibitors, siRNAs and corresponding negative controls were synthesized by GenScript (Nanjing, China). The siRNAs targeting Reck sequences are: 5′ GAACATCCTTTAGTATTGA 3′. Hiperfect transfection reagent (Qiagen Technologies) was used for cell transfection following the manufacturer’s instruction. A final concentration of 30 nM of small RNA oligos was used in all *in vitro* assays.

### First Strand cDNA Preparation and Real-Time PCR

Total RNAs were extracted by using Trizol reagent. 500 ng of total RNA was used for reverse transcription for miRNAs determination by using the M&G miRNA Reverse Transcription kit (miRGenes, Shanghai, China) according to the manufacturer’s instruction. 0.5–1.0 μg of total RNA was used for reverse transcription for mRNA determination by using iScript cDNA synthesis kit (Bio Rad, United States) Universal SYBR qPCR Master Mix and Applied Biosystems QuantStudio 6 (Applied Biosystem, Thermo Fisher Scientific) were used for real-time PCR assays. β-actin and GAPDH were used for mRNA normalization, and 5s rRNA was used for miRNA normalization. Forward primer sequences for miR-221: 5′ AGCUACAUUGUCUGCUGGGUUU 3′; miR-222: 5′ AGCUACAUCUGGCUACUGGG 3′; 5s rRNA, 5′ AGTACTTGGATGGGAGACCG 3′. All primer sequences for mRNAs are available upon request.

### Western Blot

50 μg of cell lysates was applied to 8–10% sodium dodecyl sulfate–polyacrylamide gel electrophoresis (SDS/PAGE) for protein separation. The primary antibodies (1:2,000) was used including OCT4 (2750S, Cell Signaling Technology), NANOG (4903S, Cell Signaling Technology), Notch1 (sc-373891, Santa Cruz, United States), GAPDH (sc-47724, Santa Cruz). Reck (sc-373929, Santa Cruz), NICD (sc-373891, Santa Cruz), h-TERT (sc-377511, Santa Cruz), HRP-linked anti-rabbit IgG (7074S, Cell Signaling Technology) and HRP-linked anti-mouse IgG (7076S, Cell Signaling Technology) were used as secondary antibodies (1:3,000).

### Colony Formation Assay

2,000 cells per well were seeded in a 12-well plate and cultured under regular condition for 7–10 days until visible colonies were formed. 4% paraformaldehyde was used to fix the colonies, and 0.5% crystal violet solution was used for staining. The colonies with diameter over 40 μm were counted under microscope for quantitative analysis.

### Sphere Formation Assay

Cancer cells were plated in 12-well ultra-low adherent cell culture plate (Corning, United States) at a density of 2,000 cells/well, and cultured in the serum-free medium containing DMEM/F12 with 1×B27 supplement (Invitrogen, United States), 20 ng/mL human epidermal growth factor (EGF) (Sigma, United States), and 20 ng/ml of human basic fibroblast growth factor (bFGF) (R&D Systems, United States) for 5 days without disturbing.

### Luciferase Reporter Assay

pGL-3 luciferase reporter vectors carrying either wide type (WT) or miR-221/222 binding site-mutated (MU) 3′UTR of Reck were co-transfected into 293T cells with RL-TK control vector (Renilla) and miR-221/222 mimics in a 24-well plate. After 18-h culturing, Dual-Luciferase Reporter Assay kit (Promega, United States) was used to measure the luciferase activities.

### Statistical Analysis

Data are presented as mean ± SEM unless otherwise stated. Statistical significance was determined by standard two-tailed student’s t-test followed by least-significant difference (LSD). *P* < 0.05 was considered statistically significant.

## Results

### Upregulation of miR-221/222 in the CD133^+^ CSCs in NSCLC

**FIGURE 1:**
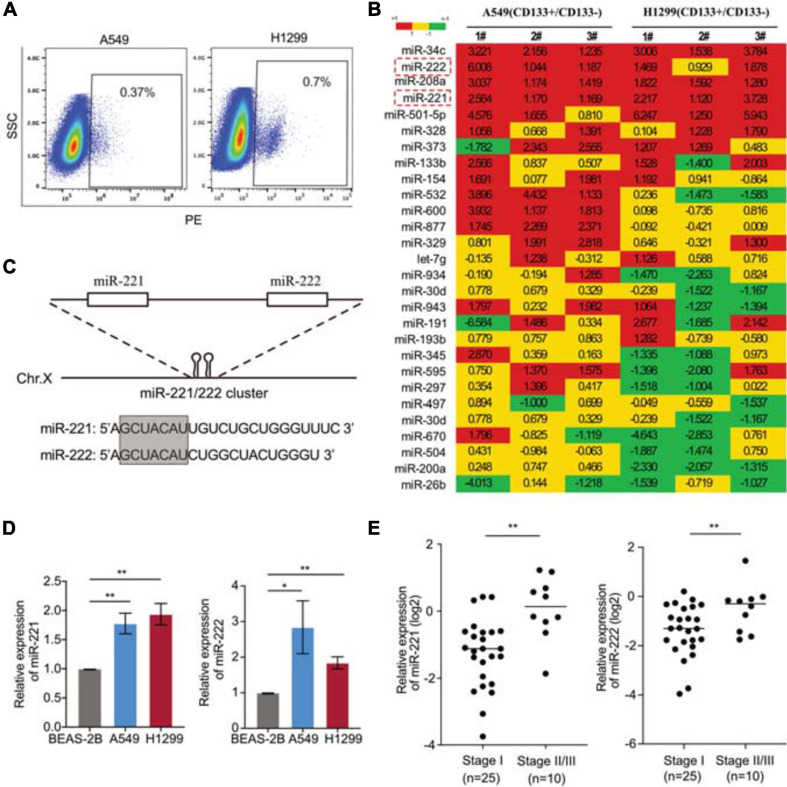
Upregulation of miR-221/222 in the CD133^+^ lung cancer stem cells. **(A)** CD133^+^ CSC isolation from A549 and H1299 cells using FACS analysis. **(B)** miRNA screening analysis on the CD133^+^ CSC subpopulation, compared with matched CD133^–^ cells. **(C)** Chromosomal structure of the miR-221/222 cluster, in which miR-221 and miR-222 share same “seed” sequence. **(D)** Upregulation of miR-221 and miR-222 in the NSCLC cell lines A549 and H1299, compared with non-tumorigenic lung epithelial cell line BEAS-2B. Data are presented as mean SEM (*N* = 3), *p < 0.05, **p < 0.01. **(E)** Higher levels of miR-221 and miR-222 in the NSCLC tumors at stages II/III, compared with the tumors at stage I. **p < 0.01.

In order to identify the key genes regulating CSCs in NSCLC, CD133^+^ cells were isolated from two NSCLC cell lines A549 and H1299 (Figure 1A). A home-made cancer-associated miRNA panel was applied for the miRNA screening analysis in the CD133^+^ CSCs, comparing to the matched CD133^–^ cells. A subset of miRNAs differentially expressed in the CSCs were identified including upregulated miR-34c, miR-208a, miR-221/222, miR-501-5p, et al., and downregulated miR-26b, miR-200a, miR-504, et al. (Figure 1B). Among those top-list of miRNAs, miR-34c and miR-501-5p have been well documented to regulate cancer cell stemness, while the function of miR-221/222 in lung CSCs remains unknown. MiR-221 and miR-222 are in a cluster sharing same “seed” sequence (Figure 1C), so we focused on the miR-221/222 cluster to determine its regulation on CSCs in NSCLC. The expression levels of miR-221 and miR-222 in NSCLC cells were detected. Both miR-221 and miR-222 showed significantly upregulation in A549 and H1299 cells compared to non-tumorigenic bronchial epithelial cell BEAS-2B (Figure 1D). The miR-221/222 analysis in tumor samples from NSCLC patients further indicated a positive correlation between the miR-221/222 levels and tumor-stage. Both miR-221 and miR-222 showed a higher level in the NSCLC tumors at stages II/III, compared with the tumor samples at stage I (Figure 1E).

### miR-221/222 Promoted Cell Proliferation in NSCLC

**FIGURE 2:**
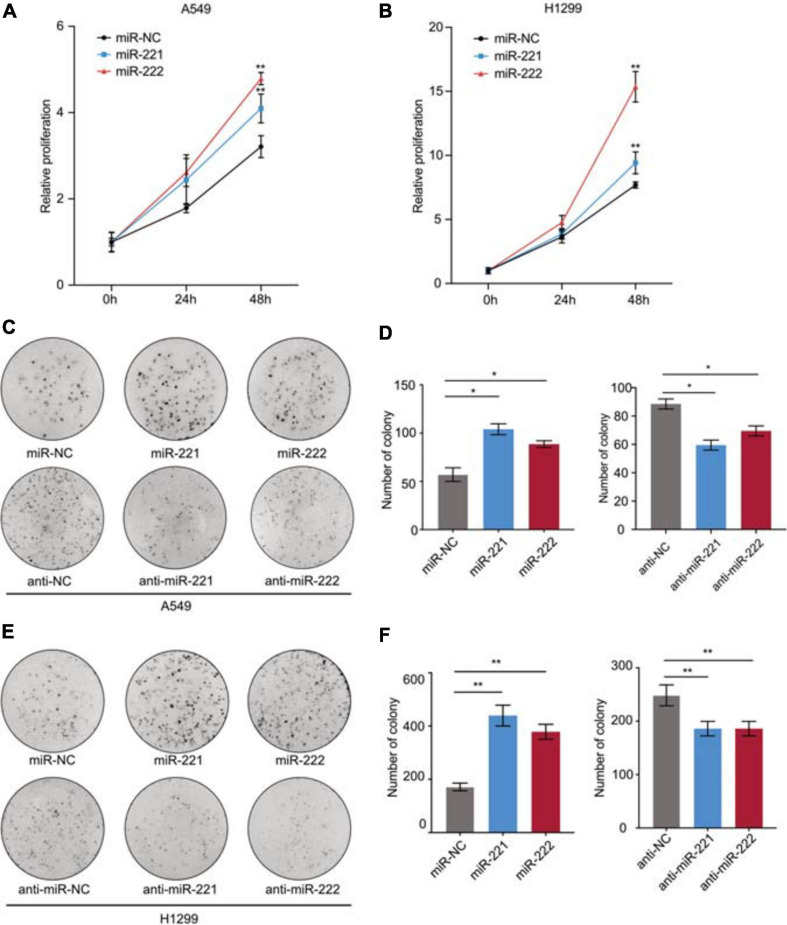
miR-221/222 promoted cell proliferation in NSCLC. **(A,B)** CCK8 assays indicating increased cell proliferation by overexpression of miR-221 and miR-222 in A549 **(A)** and H1299 **(B)** cells. **(C)** Colony formation assays indicating the positive regulation of cell proliferation by miR-221/222 in A549 cells. Both overexpression and knockdown of miR-221 or miR-222 were applied. **(D)** Quantitative analysis of panel **(C)**. **(E)** Colony formation assays indicating the positive regulation of cell proliferation by miR-221/222 in H1299 cells. Both overexpression and knockdown of miR-221 or miR-222 were applied. **(F)** Quantitative analysis of panel **(E)**. Data are presented as mean ± SEM (*N* = 3), *p < 0.05, **p < 0.01.

To determine the biological function of miR-221/222 in NSCLC, both A549 and H1299 cells were transfected with miR-221 mimic, miR-222 mimic or negative control (Supplementary Figures 1A,B), followed by CCK8 cell proliferation assay and colony formation assay. As shown in Figures 2A,B, both miR-221 and miR-222 promoted cell proliferation in NSCLC cells. In addition to miRNA overexpression, anti-miRNA inhibitor oligos targeting either miR-221 or miR-222 were applied to A549 and H1299 cells (Supplementary Figures 2A,B) followed by colony formation assay. As shown in Figures 2C–F, overexpression of miR-221 or miR-222 increased colony formation in the adhesion-independent condition. In contrast, knockdown of miR-221 or miR-222 decreased the colony formation.

### miR-221/222 Promoted the Cell Stemness in NSCLC

**FIGURE 3:**
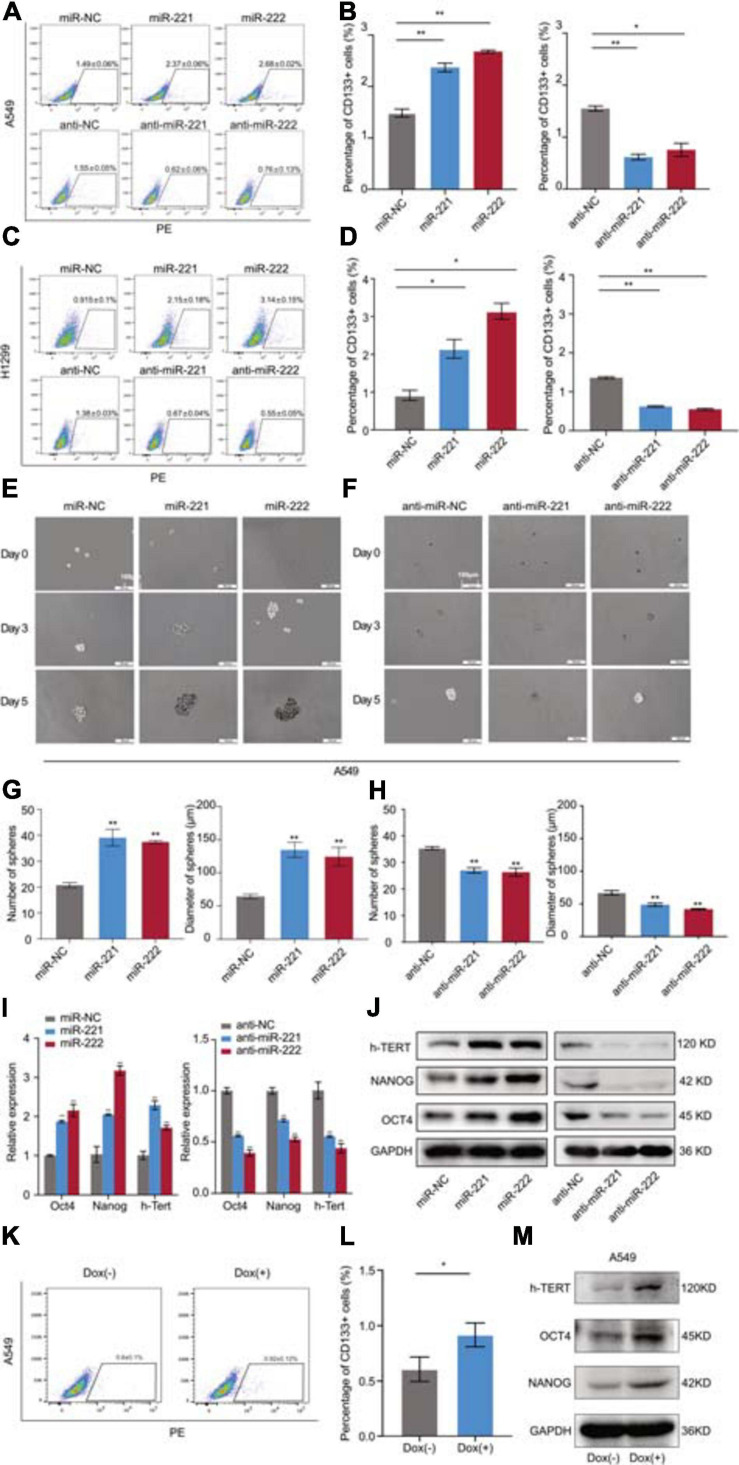
miR-221/222 promoted the cancer cell stemness in NSCLC. **(A)** Increased percentage of CD133^+^ CSCs in A549 cells after treatment with miR-221 or miR-222. Conversely, CD133^+^ CSC percentage decreased after treatment with anti-miR-221 or anti-miR-222. **(B)** Quantitative analysis of panel **(A)**. **(C)** H1299 cells were applied with same assays with panel **(A)**. **(D)** Quantitative analysis of panel **(C)**. **(E)** Sphere formation assays using A549 cells treated with miR-221 or miR-222 mimics. **(F)** Sphere formation assays using A549 cells treated with anti-miR-221 or anti-miR-222. **(G)** Quantitative analysis of the number and size of the spheres in panel **(E)**. **(H)** Quantitative analysis of the number and size of the spheres in panel **(F)**. **(I,J)** Quantitative real-time PCR **(I)** and western blot **(J)** analyses indicating the increased expression of Oct4, Nanog and h-Tert in A549 cells after treatment with miR-221 or miR-222 mimics. Consistent results were obtained after treatment with anti-miR-221 or anti-miR-222. **(K,L)** Doxycycline-induced miR-221/222 overexpression promoted CD133^+^ CSC percentage in A549 cells. **(M)** Doxycycline-induced miR-221/222 overexpression increased the expression of Oct4, Nanog, and h-Tert in A549 cells. Data are presented as mean ± SEM (*N* = 3), *p < 0.05, **p < 0.01.

Following overexpression or knockdown of miR-221/222 in A549 and H1299 cells, the changes of the CD133^+^ CSC percentage were determined by flow cytometry analysis. As shown in Figures 3A,B, overexpression of miR-221/222 in A549 cells increased CD133^+^ CSC subpopulation from 1.49% to 2.37–2.58%. In contrast, knockdown of miR-221/222 in A549 cells decreased CD133^+^ CSCs from 1.55% to 0.52–0.75%. Similar results were obtained from H1299 cells (Figures 3C,D). In addition, sphere formation assays were performed to further determine the stemness changes after overexpression (Figure 3E) or knockdown (Figure 3F) of miR-221/222 in A549 cells. Quantitative analysis indicated a positive regulation of both sphere number and sphere size by miR-221/222 (Figures 3G,H). Same assays were applied to H1299 cells and generated similar results (Supplementary Figure 3), verifying the stemness-promoting function of miR-221/222 in NSCLC. Moreover, a group of well-defined stemness genes including Oct4, Nanog, and h-Tert were examined in A549 cells by quantitative RT-PCR and western blot analyses. The results showed that miR-221/222 remarkably increased the expression of Oct4, Nanog, and h-Tert at the both mRNA (Figure 3I) and protein (Figure 3J) levels.

In order to further validate the regulation of cancer cell stemness by miR-221/222 in NSCLC, a tetracycline-inducible system was applied to miR-221/222 as shown in Supplementary Figure 4. Doxycycline-induced miR-221/222 overexpression in A549 cells significantly promoted CD133^+^ CSC percentage (Figures 3K,L), and increased the expression of Oct4, Nanog, and h-Tert as well (Figure 3M).

### Overexpression of miR-221/222 Promoted NSCLC Tumor Growth *in vivo*

**FIGURE 4:**
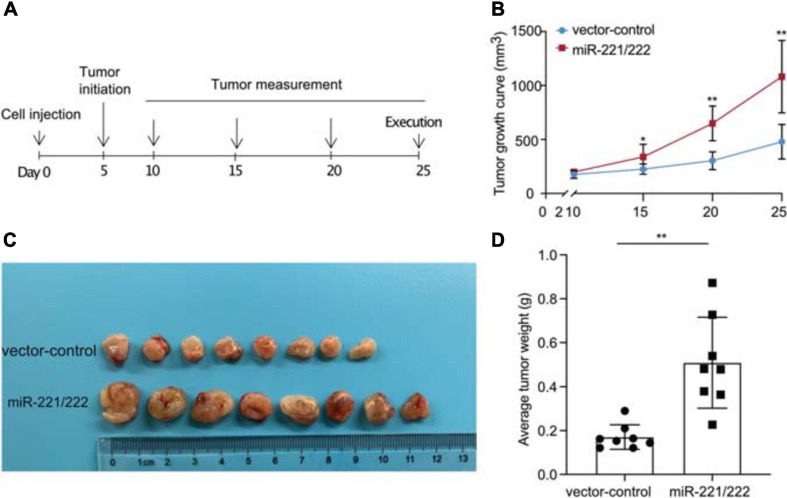
miR-221/222 promoted tumor growth in the xenograft model with NSCLC. **(A)** Schematic representation of the procedure for *in vivo* assays. **(B)** Tumor growth curves of the two groups of mice (miR-221/222-overexpressing group and control group). **(C)** Tumor images isolated from the mice. **(D)** Weight of the tumors from both groups of mice. Data are presented as mean ± SEM (*N* = 8), *p < 0.05, **p < 0.01.

In order to determine the effects of miR-221/222 on the NSCLC tumorigenesis and tumor growth *in vivo*, a NSCLC xenograft model was established by lung cancer cell xenografting into immunodeficient female nude mice. A549 cells with or without stable overexpression of miR-221/222 (Supplementary Figure 5) were applied for the xenografting, followed by the continuous tracking of tumor growth (Figure 4A). The tumor growth curves showed a significant promotion of tumor growth by overexpression of miR-221/222 (Figure 4B), which was further demonstrated by the average size and weight of the tumors on the day 25 after cell xenografting (Figures 4C,D). In addition, ki67 staining on the paraffin-embedded tumor tissue slides validated the increased tumor cell proliferation by miR-221/222 overexpression (Supplementary Figure 6).

### Activation of Notch1 Signaling by miR-221/222 *via* Suppressing the Expression of Reck

**FIGURE 5:**
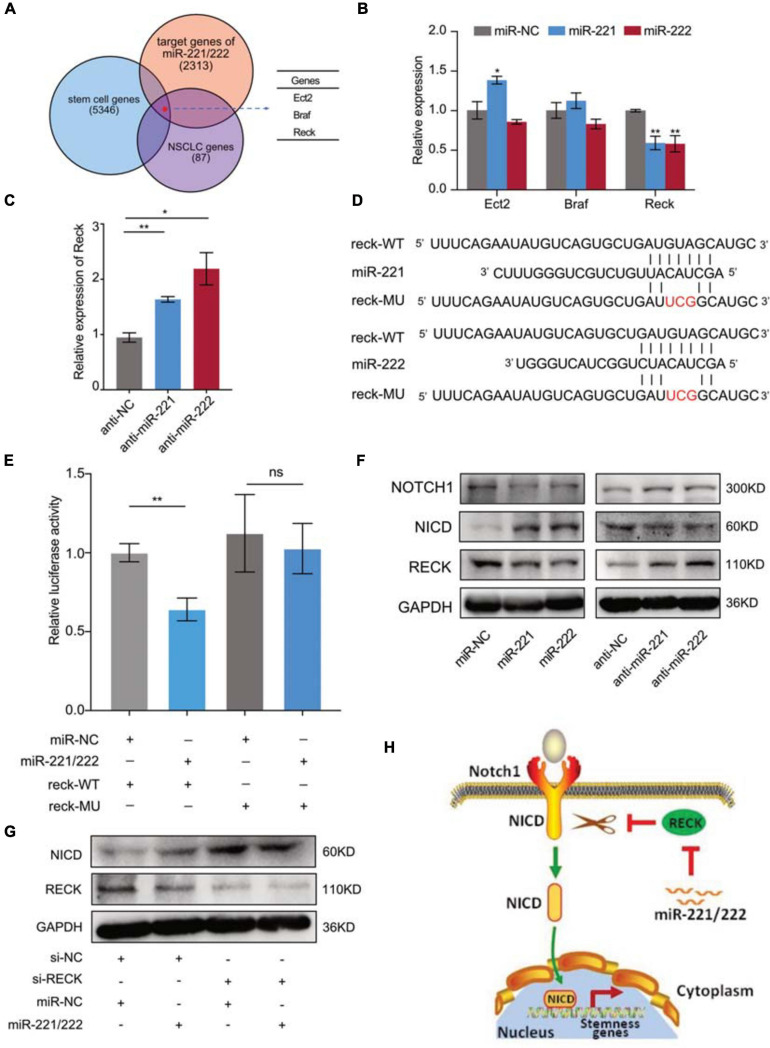
Reck gene mediated the miR-221/222-induced Notch1 signaling activation in NSCLC. **(A)** 2,313 predicted target genes of miR-221/222, 5,346 stem cell-related genes and 87 NSCLC-regulating genes overlapped 3 genes including Ect2, Braf and Reck. **(B)** Quantitative real-time PCR analysis validated downregulation of Reck by overexpression of miR-221/222 in A549 cells. **(C)** Upregulation of Reck was shown after knockdown of miR-221/222 in A549 cells. **(D)** Wild type (WT) or point mutated (MU) Reck 3′UTR were cloned into pGL-3 Luciferase reporter vector. The miR-221/222 binding site was mutated in MU. **(E)** Luciferase reporter assays indicating the direct inhibition of Reck WT 3′UTR by miR-221/222, which was attenuated by the binding-site mutation. **(F)** Western blot analyses indicating the activation of Notch1 signaling by miR-221/222 overexpression in A549 cells, including increased level of Notch1 intracellular domain (NICD) and decreased level of uncleaved Notch1, which were associated with downregulation of Reck. Consistent results were obtained in A549 cells after knockdown of miR-221/222. **(G)** Western blot analyses indicating the increase of NICD by knockdown of Reck. But miR-221/222 did not show regulation on the level of NICD in the si-Reck-treated A549 cells. **(H)** Schematic representation of the mechanism through which miR-221/222 promoted CSCs properties in NSCLC by suppressing Reck and activating Notch1 signaling. Data are presented as mean ± SEM (*N* = 3), *p < 0.05, **p < 0.01, ns means non significant.

Next we sought to determine the molecular mechanism(s) through which miR-221/222 regulates CSCs in NSCLC. Bioinformatics analyses predicted 2,313 target genes of miR-221/222, 5,346 stem cell-related genes and 87 NSCLC-regulating genes using public databases. Among them 3 genes were overlapped including Ect2, Braf and Reck (Figure 5A). Quantitative RT-PCR analysis revealed that overexpression or knockdown of miR-221/222 significantly decreased or increased Reck gene expression, respectively (Figures 5B,C), suggesting that Reck is a potential target gene of miR-221/222 in lung cancer cells. In order to verify whether Reck is a direct target of miR-221/222, luciferase reporter constructs carrying either wide type (WT) or miR-221/222-binding sites-mutated (MU) 3′UTR of Reck were co-transfected with miR-221/222 mimics into 293T cells (Figures 5D,E), and the results we obtained from this experiment strongly supported that the inhibition of Reck 3′UTR by miR-221/222, relying on their sequence complementary binding (Figure 5E).

The protein encoded by Reck is an extracellular protein with protease inhibitor-like domains with downregulation in many tumors and cells transformed by various kinds of oncogenes, functioning as a tumor suppressor. In view of cell stemness regulation by Reck by suppressing Notch1 shedding and activation in gastric cancer (Hong et al., 2014), we tested the expression changes of Reck and Notch1 in A549 cells with or without overexpression of miR-221/222. As shown in Figure 5F, both miR-221 and miR-222 reduced the protein levels of Reck and Notch1, but increased the expression level of Notch1 intracellular domain (NICD). Consistent results were obtained in A549 cells after knockdown of miR-221/222 (Figure 5F). NICD is the activated form of Notch1 after cleavage. It has been demonstrated that NICD functions as a transcriptional factor to induce cell stemness (Hong et al., 2014; Lee et al., 2016). Since Reck negatively regulates the Notch1 cleavage, and thereby suppresses the CSC properties (Hong et al., 2014), siRNA targeting Reck (si-Reck) was applied to A549 cells to test its effect on the Notch1 signaling. Significant increase of NICD was observed when Reck was knocked down by siRNA (Figure 5G). Furthermore, in the si-Reck-treated A549 cells, overexpression of miR-221/222 did not show more induction of the Notch1 activation (Figure 5G). These data suggest the requirement of Reck expression for the miR-221/222-induced Notch1 signaling activation and cell stemness promotion in NSCLC (Figure 5H).

## Discussion

Emerging evidences indicate that many genetic and epigenetic changes underlying the aggressive and destructive behavior of cancer cells are orchestrated by a small population of cancer cells called CSCs within tumor tissues (Easwaran et al., 2014). However, the understanding of the identification, biomarkers, and cellular properties of CSCs are limited and variable upon tumor types. Herein, we identified the miR-221/222 cluster with upregulation in CD133^+^ CSCs as an essential positive regulator promoting the stemness of NSCLC cells, which are closely associated with tumorigenesis and tumor growth in NSCLC. Further, we demonstrated that a Reck-Notch1 signaling mediates the miR-221/222-induced increase of CSC properties in NSCLC.

It has been reported that Reck gene inhibits cell migration, invasion and angiogenesis by negatively regulating matrix metalloproteinases (MMPs) and a disintegrin and metalloproteinase 10 (ADAM10) (Hong et al., 2014). In gastric cancer, ectopic expression of Reck gene suppressed the expression of stemness genes and the sphere formation and sphere size of CD133^+^ CSCs (Hong et al., 2014). In the current study, we are the first to demonstrate that Reck gene, a direct target of miR-221/222, is an important mediator for the miR-221/222-induced CSC properties, and verify the involvement of Reck gene in the epigenetic regulation of CSCs. Our previous work has demonstrated the oncogenic function of miR-221/222 in regulating the cellular migration, invasion, CSCs and drug-resistance in triple negative breast cancer (Li et al., 2014, 2020). However, the regulatory function and mechanism of miR-221/222 in lung CSCs remain unclear. Garofalo et al. reported in 2009 that the overexpression of miR-221/222 in aggressive NSCLC cells induced TRAIL resistance and enhanced cellular migration by targeting Pten and Timp3 (Garofalo et al., 2009). Besides this, there is no other report regarding the effect of miR-221/222 on NSCLC cells, especially CSCs.

In this study, in order to explore the fundamental mechanism(s) by which how miR-221/222 influences CSCs properties in NSCLC, we decided to look at Notch1 signaling since Reck functions as a tumor suppressor by suppressing Notch1 shedding and activation (Hong et al., 2014). Notch1 signaling has been shown to be critical for cell proliferation, cell fate specification and CSC self-renewal (Chiba, 2006; Venkatesh et al., 2018). It is also reported that a cleaved form of Notch1, NICD promoted the self-renewal capacity of CSCs in head and neck squamous cell carcinoma by activating sphere formation and increasing the expression of stem cell markers (Lee et al., 2016). Here in our study, we found that miR-221/222 downregulates Reck but increases the NICD level, suggesting that miR-221/222 enhances stemness of NSCLC cells mostly through activating Notch 1 signaling. However, how miR-221/222-Reck signaling promotes the cleavage of Notch1 into NICD needs to be further investigated. CSCs are one of the sources for cancer initiation, development and metastasis. Emerging evidences show that targeting Notch signaling pathway of CSCs represents a promising therapeutic strategy to treat cancer (Zhao et al., 2016; Venkatesh et al., 2018). Our *in vivo* study clearly showed that overexpression of miR-221/222 significantly enhanced tumor growth, providing an evidence to target miR-221/222 as an upstream regulator of Notch1 signaling in regulating CSCs and achieving potential therapeutic effects on NSCLC.

Many transcriptional factors and signaling pathways are involved in regulation of cancer cell dormancy and cancer stem cells (Talukdar et al., 2019). For example, upregulation of Forkhead box M1 (FOXM1) in cancer stem cells is associated with poor prognosis in a variety of tumor types (Zhang et al., 2017). In breast cancer, FOXM1 was found to promote the activity of YAP1 and maintain the cancer cell stemness (Sun et al., 2020). The crosstalk between YAP/TAZ and Notch signaling influences cell self-renewal, stem cell differentiation, cell fate decisions, epithelial-stromal interactions, inflammation, morphogenesis, and large-scale gene oscillations (Totaro et al., 2018). Overexpression of miR-222 has been reported to induce activation of YAP-TEAD1 signaling and promote cell proliferation and invasion in gastric cancer cells (Li et al., 2015). Although a miR-221/222 binding site was predicted in the 3′-UTR of YAP1, it remains unknown whether miR-221/222 regulate YAP1 expression in NSCLC. Nevertheless, the finding in the current study of the mir-221/222-RECK-Notch signaling adds a node to the regulatory network of CSCs in NSCLC.

Epidermal growth factor receptor (EGFR) mutation can lead to pathogenesis in NSCLC (Hsu et al., 2018). EGFR inhibitors including gefitinib and erlotinib have been applied for the first-line treatment of EGFR-mutant NSCLC (Yang et al., 2017). Targeted inhibition of miR-221/222 has been demonstrated to induce cancer cell sensitivity to TRAIL, gefitinib and erlotinib (Garofalo et al., 2009, 2011; Jang et al., 2016). In view of the close correlation between CSCs and chemoresistance, the findings in our study may provide a clue to interpret the miR-221/222-induced chemoresistance in NSCLC.

In summary, our findings indicate that miR-221/222 functions as a novel critical regulator of CSCs in NSCLC through suppressing Reck and activating Notch1 signaling. The miR-221/222-Reck-Notch1 axis not only represents a vital epigenetic mechanism in regulating CSC self-renewal and maintenance, but also provides potential targets for inhibition of CSCs in treatment of SCLC.

## Data Availability Statement

The original contributions presented in the study are included in the article/Supplementary Material, further inquiries can be directed to the corresponding author/s.

## Ethics Statement

The studies involving human participants were reviewed and approved by the Institutional Review Board (IRB) of Tongji University Shanghai East Hospital. The patients/participants provided their written informed consent to participate in this study. The animal study was reviewed and approved by Experimental Animal Center of Tongji University School of Medicine.

## Author Contributions

QW, QL, and ZY designed the project and wrote the manuscript. YG and QZ did the data analysis. YG, GW, ZW, LQ, XD, PL, WM, DL, and YLi performed the cell and molecular biology experiments. YG, JL, ZR, and YuL did the animal study. All authors contributed to the article and approved the submitted version.

## Conflict of Interest

The authors declare that the research was conducted in the absence of any commercial or financial relationships that could be construed as a potential conflict of interest.
