# Supplementary material for: Reck-Notch1 Signaling Mediates miR-221/222 Regulation of Lung Cancer Stem Cells in NSCLC
Source: Front Cell Dev Biol. 2021 Apr 20;9:663279. doi: 10.3389/fcell.2021.663279 (PMC8093830; doi:10.3389/fcell.2021.663279)

Supplemental Figure Legends

Supplemental Figure S1. Validation of miR-221/222 overexpression. A: Validation of the miR-221 and miR-222 overexpression in A549 cells. B: Validation of the miR-221 and miR-222 overexpression in H1299 cells. Data are presented as mean ± SEM (N=3), **p<0.01.

Supplemental Figure S2. Validation of miR-221/222 knockdown. A: Validation of the miR-221 and miR-222 knockdown in A549 cells. B: Validation of the miR-221 and miR-222 knockdown in H1299 cells. Data are presented as mean ± SEM (N=3), **p<0.01.

Supplemental Figure S3. Figure 3. miR-221/222 promoted the sphere formation in H1299 cells. A: Sphere formation assays using H1299 cells treated with miR-221 or miR-222 mimics. B: Sphere formation assays using H1299 cells treated with anti-miR-221 or anti-miR-222. C: Quantitative analysis of the number and size of the spheres in A. D: Quantitative analysis of the number and size of the spheres in B. Data are presented as mean ± SEM (N=3), **p<0.01.

Supplemental Figure S4. Validation of miR-221/222 overexpression by doxycycline treatment in the A549 cells transduced with tet-inducible vectors . Data are presented as mean ± SEM (N=3), *p<0.05.

Supplemental Figure S5. Validation of miR-221/222 overexpression in the A549 cells transduced with a lentivirus vector carrying pre-miR-221/222 sequence. Data are presented as mean ± SEM (N=3), **p<0.01.

Supplemental Figure S6. Ki67 staining on the representative slides of paraffin-embedded tumor tissues from the mice transplanted with either miR-221/222-overexpressing A549 or vector control-A549 cells.

Supplemental Figure S1

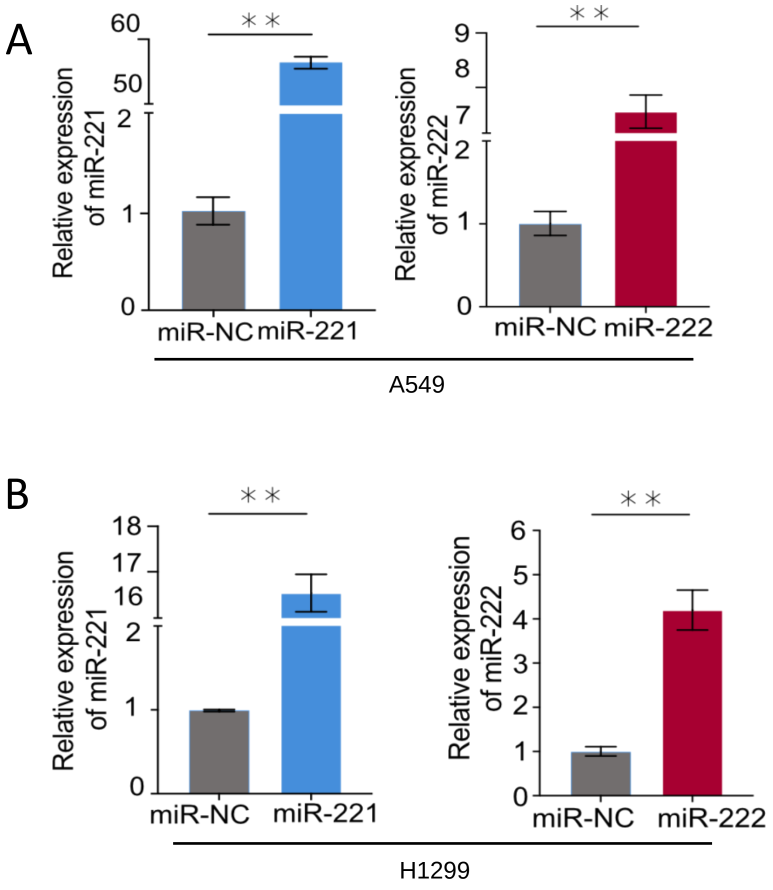

Supplemental Figure S2

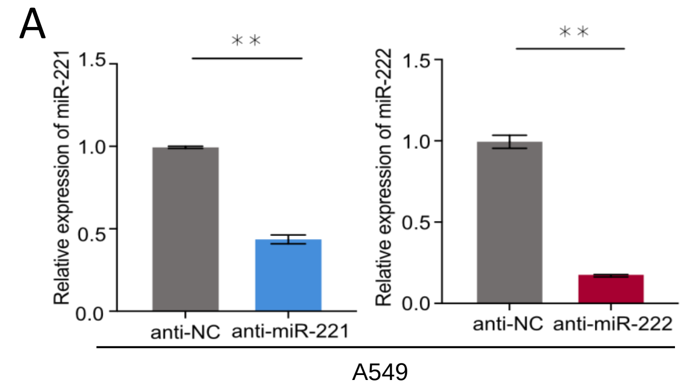

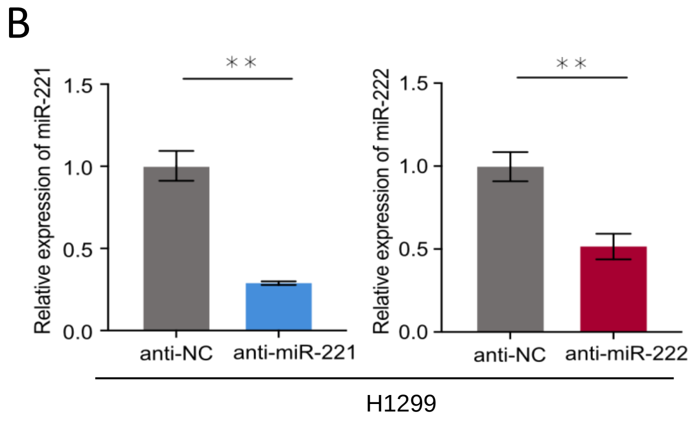

Supplemental Figure S3

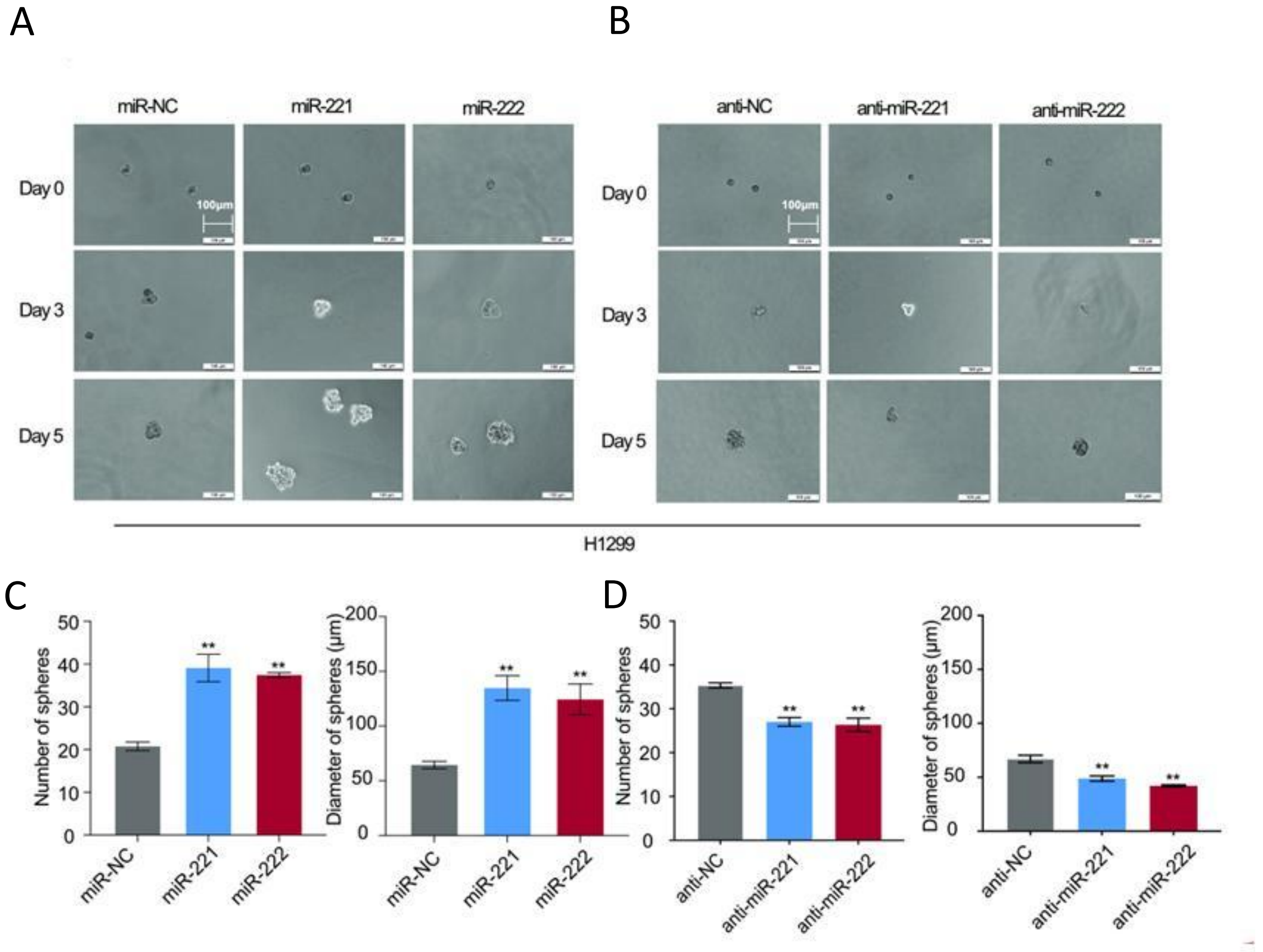

Supplemental Figure S4

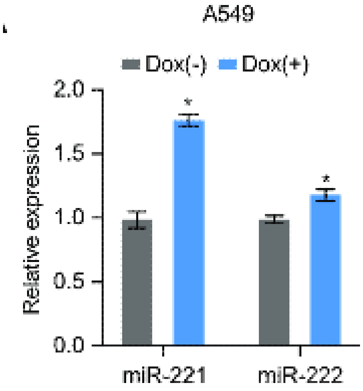

Supplemental Figure S5

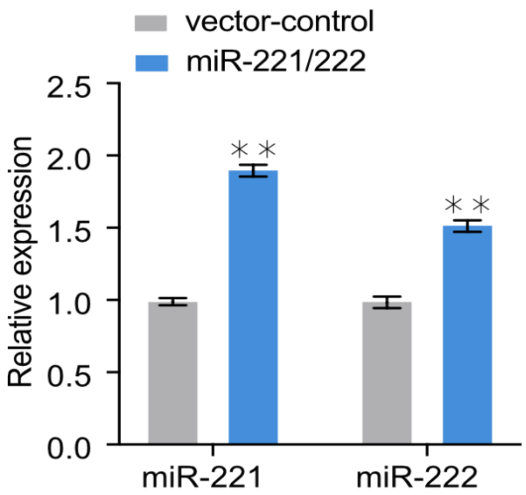

Supplemental Figure S6

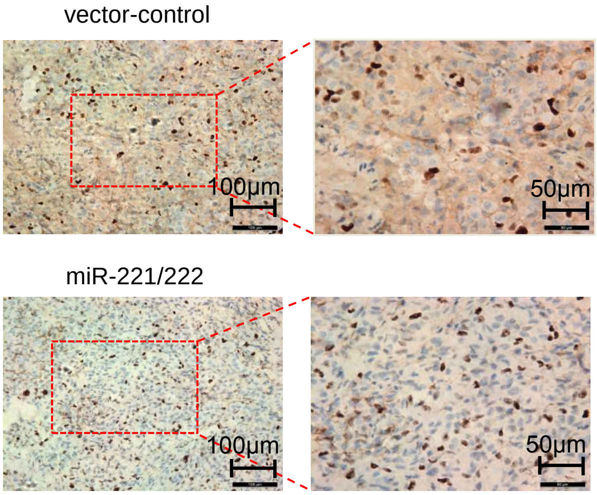

Supplement: Supplementary file 1 [file Data_Sheet_1.PDF]
